# A Novel Nonsense *INS* Mutation Causes Inefficient Preproinsulin Translocation Into the Endoplasmic Reticulum

**DOI:** 10.3389/fendo.2021.774634

**Published:** 2022-01-05

**Authors:** Ying Yang, Hua Shu, Jingxin Hu, Lei Li, Jianyu Wang, Tingting Chen, Jinyang Zhen, Jinhong Sun, Wenli Feng, Yi Xiong, Yumeng Huang, Xin Li, Kai Zhang, Zhenqian Fan, Hui Guo, Ming Liu

**Affiliations:** ^1^Department of Endocrinology and Metabolism, Tianjin Medical University General Hospital, Tianjin, China; ^2^Department of Endocrinology, The Second Part of Jilin University First Hospital, Jilin, China; ^3^Division of Metabolism, Endocrinology and Diabetes, University of Michigan Medical School, Ann Arbor, MI, United States; ^4^Department of Technology Services, RSR Tianjin Biotech Co., Tianjin, China; ^5^Department of Endocrinology and Metabolism, The Second Hospital of Tianjin Medical University, Tianjin, China

**Keywords:** *INS* gene, mutation, diabetes, preproinsulin, translocation

## Abstract

Preproinsulin (PPI) translocation across the membrane of the endoplasmic reticulum (ER) is the first and critical step of insulin biosynthesis. Inefficient PPI translocation caused by signal peptide (SP) mutations can lead to β-cell failure and diabetes. However, the effect of proinsulin domain on the efficiency of PPI translocation remains unknown. With whole exome sequencing, we identified a novel *INS* nonsense mutation resulting in an early termination at the 46th residue of PPI (PPI-R46X) in two unrelated patients with early-onset diabetes. We examined biological behaviors of the mutant and compared them to that of an established neonatal diabetes causing mutant PPI-C96Y. Although both mutants were retained in the cells, unlike C96Y, R46X did not induce ER stress or form abnormal disulfide-linked proinsulin complexes. More importantly, R46X did not interact with co-expressed wild-type (WT) proinsulin in the ER, and did not impair proinsulin-WT folding, trafficking, and insulin production. Metabolic labeling experiments established that, despite with an intact SP, R46X failed to be efficiently translocated into the ER, suggesting that proinsulin domain downstream of SP plays an important unrecognized role in PPI translocation across the ER membrane. The study not only expends the list of *INS* mutations associated with diabetes, but also provides genetic and biological evidence underlying the regulation mechanism of PPI translocation.

## Introduction

Insulin is an essential hormone for maintaining glucose homeostasis of the body. In pancreatic β-cells, the insulin biosynthesis starts from its precursor, preproinsulin (PPI), which is composed of the N-terminal signal peptide (SP) followed by the C-terminal proinsulin domain. Newly synthesized PPI driven by its SP translocates across the membrane of the endoplasmic reticulum (ER) through the Sec61 translocon. Upon delivery into the ER, SP of PPI is cleaved by signal peptidase at the luminal side of the ER membrane, forming another insulin precursor, proinsulin (PI) (1). In the oxidized ER lumen, PI undergoes rapid oxidative folding, forming three highly conserved disulfide bonds (B7-A7, B19-A20, and A6-A11). Well-folded PI exits from the ER and traffics through the Golgi to the secretory granules where it is processed by prohormone convertase (PC1/3 and PC2) and carboxypeptidase E (CPE), forming mature insulin and C-peptide (2–4). It takes approximately 30–150 min to finish these intracellular processes. Among these events, the earliest step during which PPI translocation into the ER had been long thought to be very efficient and exclusively through the signal recognition particle (SRP)-dependent co-translational translocation (5). However, recent evidence indicates that, as a small secretory protein with a suboptimal signal sequence, the fully synthesized PPI may not be efficiently recognized by SRP and co-translationally translocated into the ER. SRP independent post-translational translocation then functions as an important backup to enhance PPI translocation (4, 6, 7). The pathophysiological significance of inefficient PPI translocation is highlighted by recent evidence showing that deficiency of TRAPα [translocon associated protein alpha, a type 2 diabetes associated gene (8)], TRAPβ, and/or TRAPδ impairs PPI translocation and insulin production (9, 10), and also by the discoveries of PPI SP mutations that impair PPI translocation causing β-cell failure and diabetes in humans (2, 11–14).

In addition to the PPI SP mutations, at least 70 *INS* gene mutations located in the proinsulin domain have been reported to be associated with diabetes in humans (15–20). The majority of these mutations impair proinsulin oxidative folding in the ER, causing proinsulin misfolding, and decreasing insulin production (21–24). However, no *INS* mutation located in the proinsulin domain has been reported to affect translocation of PPI. Herein, we identified a novel nonsense *INS* mutation causing an early termination at the 46th residue of PPI (PPI-R46X) in two unrelated patients with early-onset diabetes. We found that, unlike other proinsulin domain mutations, R46X did not appear to induce strong ER stress or form abnormal disulfide-linked proinsulin complexes (DLPC) with co-expressed wild-type (WT) proinsulin in the ER. Metabolic labeling experiments established that, despite with an intact SP, R46X failed to be efficiently translocated into the ER, suggesting an important unrecognized role of proinsulin domain in determining the efficiency of PPI translocation. Genetic testing, pedigree analysis, and clinical presentations revealed a broad spectrum of diabetes phenotypes among the members carrying R46X in the two families, suggesting that other genetic and environmental factors may contribute to actual clinical presentations associated with R46X. This study not only expends the list of *INS* mutations associated with diabetes, but also provides genetic and biological evidence underlying the regulation mechanism of PPI translocation.

## Materials and Methods

### Patients

Two unrelated patients with early-onset diabetes were referred for genetic testing of the monogenic diabetes. The clinical information was collected and recorded by the Tianjin Medical University General Hospital and the second Part of Jilin University First Hospital. The clinical information of these two probands and their family members is described in **Tables 1**–**3**. An informed consent has been signed by patients and their families. The study was approved by Tianjin Medical University General Hospital Ethics Committee (No. IRB2017-047-01).

**Table 1:** Clinical characterization of the patients with INS-R46X mutation.

		Proband1	Proband2
**Gender**		Female	Female
**At diagnosis**	Age (years)	17	8
	BMI (kg/m^2^)	24.8	20.9
	FBG (mmol/L)	9.4	14
	C-Peptide (ng/ml)	3.3	2.06
	HbA1c (%, mmol/mol)	7.8, 62	13, 119
	Symptoms of diabetes	polydipsia, polyphagia, polyuria	No
	75g-OGTT		
	Glucose (mmol/L)	9.16 (0 h)–18.13 (1 h)–18.01 (2 h)	6.1 (0 h)–15.7 (1 h)–15.4 (2 h)
	C-peptide (ng/ml)	3.3 (0 h)–5.02 (1 h)–5.8 (2 h)	1.1 (0 h)–4.45 (1 h)–4.18 (2 h)
	GADA	(-)	(-)
	ICA	(-)	(-)
	IA-2A	(-)	(-)
	ZnT8A	(-)	(-)
	Treatment	Insulin 0.71 (U/kg/day)	Insulin 0.73 (U/kg/day)
**At present**	Age (years)	20	11
	FBG (mmol/L)	7.76	16.7
	Insulin (mU/L)	28.26	26.88
	C-Peptide (ng/ml)	-	0.94
	HbA1c (%, mmol/mol)	7.1, 54.1	14.3, 133
	Treatment	Metformin	Insulin 1.87 (U/kg/day)
**Family history**		Maternal uncle had diabetes	Maternal grandmother, maternal uncle, mother, and older sister had diabetes

BMI, body mass index; FBG, fasting blood glucose; GADA, glutamate decarboxylase antibody; ICA, islet cell antibody; IA-2A, protein tyrosine phosphatase-like protein antigen; ZnT8A, Zinc transporter 8 antibody.

**Table 2:** Clinical characterization of the subjects with INS-R46X mutation from family 1.

	Mother	Brother
**Age (years)**	53	28
**BMI (kg/m^2^)**	24.2	33.6
**FBG (mmol/L)**	5.4	5.7
**C-Peptide (ng/ml)**	2.26	4.85
**HbA1c (%, mmol/mol)**	5.9, 41	5.7, 38.8
**75g-OGTT**		
**Glucose (mmol/L)**	5.36 (0 h)–8.40 (1 h)–7.88 (2 h)	5.73 (0 h)–10.5 (1 h)–8.03 (2 h)
**Insulin (mU/L)**	7.4 (0 h)–28.7 (1 h)–31.0 (2 h)	23.4 (0 h)–108.9 (1 h)–135.4 (2 h)
**C-peptide(ng/ml)**	2.26 (0 h)–6.17 (1 h)–7.9 (2 h)	4.85 (0 h)–11.65 (1 h)–13.79 (2 h)

BMI, body mass index; FBG, fasting blood glucose.

**Table 3:** Clinical information of patients with diabetes from family 2.

		Elder sister	Mother	Maternal uncle
**At diagnosis**	Age (years)	20	40	40
	BMI (kg/m²)	29.4	25.3	21.3
	FBG (mmol/L)	17	17	10
	C-peptide (ng/ml)	1.17	1.77	NA
	HbA1c (%, mmol/mol)	15.1, 142	12.9, 117	NA
	Symptoms of diabetes	Polydipsia, polyuria	Polydipsia, polyuria	Polydipsia, polyphagia, polyuria, loss of weight
	Treatment	Insulin 0.61 (U/kg/day)	Metformin	Metformin
**At present**	Age (years)	24	44	46
	FBG (mmol/L)	18.5	17	11
	Treatment	Insulin 0.61 (U/kg/day)	Metformin	Metformin

BMI, body mass index; FBG, fasting blood glucose; NA, not available.

### Genetic Testing and Analyses

Targeted gene capture followed by whole exome sequencing with massively parallel next-generation sequencing (NGS) was performed with the genomic DNA extracted from white blood cells. The identified *INS* gene mutation was confirmed by Sanger sequencing with the primer: Forward 5’-TCAGCCCTGCCTGTCTCC-3’, Reverse 5’-AAAAGTGCACCTGACCCCCTG-3’. The members of the two families of two probands were recruited and tested for the mutation by Sanger sequencing.

### Reagents and Antibodies

Lipofectamine 2000, Lipofectamine 3000, and 4–12% NuPage gel were purchased from Invitrogen (Carlsbad, CA, USA). Protein A-Agarose was from Santa Cruz Biotechnology (Dallas, TX, USA). Guinea pig anti-insulin was from Merck Millipore (Billerica, MA, USA). Rabbit anti-Human proinsulin was from Biogot (Nanjing, China). Rabbit anti-Myc antibody was from Immunology Consultants Laboratories (Portland, OR, USA). Mouse anti-Hsp90 and anti-GFP antibody were from Sungene Biotech (Tianjin, China). Goat anti-guinea pig IgG Alexa Fluor 488 and goat anti-rabbit IgG Alexa Fluor 555 was bought from Invitrogen (Carlsbad, CA, USA). Enhanced chemiluminescence Western blotting substrate was from Millipore (Billerica, MA, USA). Trans^35^S label and pure ^35^S-methionine were from PerkinElmer (Waltham, MA, USA). Met/Cys-deficient Dulbecco’s modified Eagle’s medium (DMEM) was from Invitrogen (Thermo Fisher, Waltham, MA, USA).

### Construction of Plasmids Encoding Preproinsulin Wild-Type and Mutants

The plasmids encoding human PPI WT with GFP-tag in the C-peptide were described previously (6). The DNA sequences of human PPI WT with or without Myc-tag in C-peptide and mutants (C96Y, R46X, G44R, P52L, H29T, and H29T/R46X) with Myc-tag were synthesized and introduced into the vector pcDNA3.1 (Tsingke Biological Technology, Beijing, China). All mutations were confirmed by DNA sequencing with primers: CMV-F 5’-CGCAAATGGGCGGTAGGCGTG-3’, BGH-R 5’-TAGAAGGCACAGTCGAGG-3’.

### Cell Culture and Transfection, ^35^S-Met/Cys Metabolic Labeling, and Immunoprecipitation/Co-Immunoprecipitation

Human embryonic kidney 293T (HEK293T) cells and INS1 rat insulinoma cells were purchased from ATCC (Manassas, VA, USA). 293T cells were cultured in Dulbecco’s Modified Eagle Medium (DMEM) with 10% fetal bovine serum (FBS), penicillin (100 units/ml), and streptomycin (100 μg/ml). The INS1 cells were cultured in RPMI 1640 (containing D-Glucose 2000 mg/L) supplemented with 10% FBS and 0.05 mM 2-mercaptoethanol (Sigma, St Louis, MO, USA). For the transfection, 293T cells were seeded into 12-well plates 1 day before the transfection. For each well, a total of 1 μg of plasmid DNA was transfected using Lipofectamine 2000. For metabolic labeling experiments, at 48 h post-transfection, the cells were pulse labeled with ^35^S-Met or ^35^S-(Met+Cys) and chased for the times indicated. The cells were harvested and lysed. Trichloroacetic acid (TCA)-precipitable counts were performed to normalize the amount of the total proteins among samples. The cell lysates were immunoprecipitated with anti-insulin antibody overnight at 4°C, and analyzed using 4%–12% NuPage gel under reducing condition as previously described (25). For co-immunoprecipitation (Co-IP), the cells were lysed with co-IP buffer (100 mM NaCl, 25 mM Tris, pH 7.0, 0.1% Triton X-100, 5 mM EDTA, and protease inhibitors mixture). Ninety percent of the total lysates were immunoprecipitated with anti-GFP at 4°C for 3 h. The remaining 10% of the total lysates were used to determine the expression levels of Myc-tagged and GFP-tagged PPI WT and Myc-tagged mutants. The immunoprecipitates and the total lysates were resolved using 4%–12% NuPage gel under reducing conditions followed by immunoblotted with anti-Myc and anti-proinsulin antibodies as indicated.

### Immunofluorescence Assay

An immunofluorescence assay was employed in INS832/13 cells transfected with plasmids encoding Myc-tagged PPI WT or mutants (C96Y and R46X). Briefly, transfected INS832/13 cells grown on coverslips were fixed with 4% paraformaldehyde for 30 min at room temperature, followed by permeabilization with 0.2% Triton for an additional 15 min and then blocking with 5% BSA. The cell samples were incubated with primary antibodies (Anti-Myc 1:3000 dilution, anti-insulin 1:5,000 dilution) followed by appropriate secondary antibodies conjugated with different fluor as indicated. Immunofluorescence images were acquired by using Axio Imager M2 (ZEISS, Baden-Württemberg, Germany).

### Statistical Analysis

All data were processed with GraphPad Prism 8 and presented as means ± SEM. One-way ANOVA test was used to determine significance between the groups. A *p*-value < 0.05 was considered as statistically significant.

## Results

### Identification of a Novel Nonsense *INS* Mutation R46X Associated With Diabetes in Two Unrelated Patients

**Figure 1:**
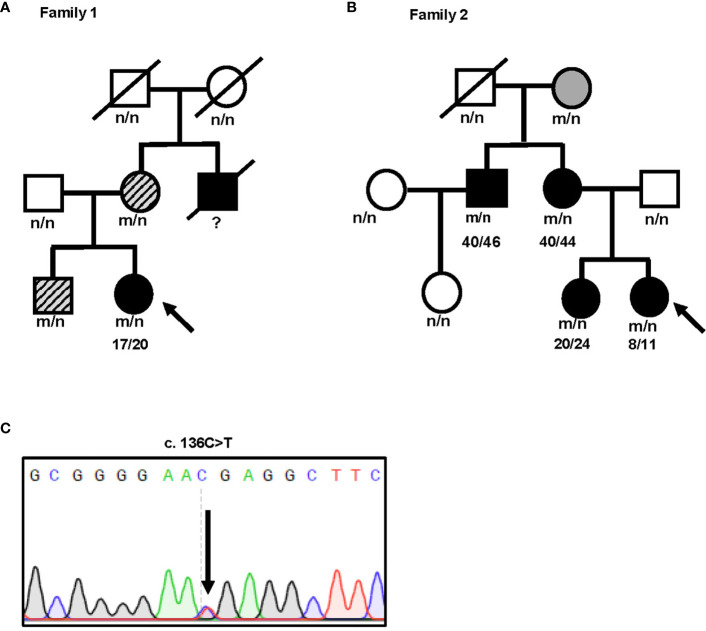
Identification of a novel nonsense *INS* mutation R46X associated with diabetes in two unrelated patients. **(A, B)** Pedigree and genotypes of two families with early-onset diabetes. Subjects carrying R46X mutation without diabetes are shown in gray. Subjects carrying R46X mutation but just diagnosed as impaired glucose regulation are shown in gray with slash. Subjects with diabetes are shown in black. Black arrow indicates the probands. n, normal allele; m, mutant allele. **(C)** DNA sequences of the *INS* mutation c.136 C>T found in both probands.

We identified an *INS* gene mutation (NM_001042376.2: p. Arg46*/c.136C>T) in two unrelated patients with early-onset diabetes (**Figure 1**). Whole genome sequencing indicated that no additional known gene mutations associated with monogenic diabetes was found. Their clinical features at diagnosis of diabetes are shown in **Table 1**. The proband 1 was diagnosed with diabetes at the age of 17 due to the symptoms of polyuria, polydipsia, polyphagia with elevated fasting blood glucose (9.4 mmol/L), and diabetic ketosis. Her BMI was 24.8 kg/m^2^ and the common T1DM autoantibodies (glutamate decarboxylase antibody [GADA], islet cell antibody [ICA], protein tyrosine phosphatase-like protein antigen [IA-2A], and Zinc transporter 8 antibody [ZnT8A]) were all negative. She was treated with insulin (0.71 U/kg/day) at the time of diagnosis. Self-monitoring of blood glucose (SMBG) showed that her fasting blood glucose (FBG) was 4–5 mmol/L and 2 h postprandial blood glucose (2hPBG) was 6–9 mmol/L, suggesting that her diabetes was well controlled. Subsequently 1 year later, she withdrew insulin without doctor’s advice and only took metformin 1.5 g/day. Her HbA1c was 7.1% at the last visit. Notably, the proband’s mother and older brother were found to carry the same mutation but only had impaired glucose regulation (**Figure 1A** and **Table 2**). Her maternal uncle was diagnosed with diabetes at the age of 40. Unfortunately, his blood sample was unavailable for genetic testing because he has passed away. The second proband was diagnosed with diabetes when she was 8 years old. She was accidentally found to have hyperglycemia when she had an upper respiratory tract infection. At the time of diabetes diagnosis, her BMI was 20.9 kg/m^2^ and HbA1c was 13% without typical symptoms of diabetes. She was initiated insulin therapy (0.73 U/kg/day). However, the patient adherence was low and her blood glucose was poorly controlled even with a high dose of insulin therapy (1.87 U/kg/day). Except for her maternal grandmother whose fasting blood glucose were 6–7 mmol/L, her family members carrying this mutation were all diagnosed with diabetes at various onset ages (**Figure 1B** and **Table 3**).

### R46X Mutant Impaired Secretion of the Mutant but Did Not Affect Secretion or Production of Bystander Proinsulin-WT, nor Did It Impair Production of Endogenous Insulin

**Figure 2:**
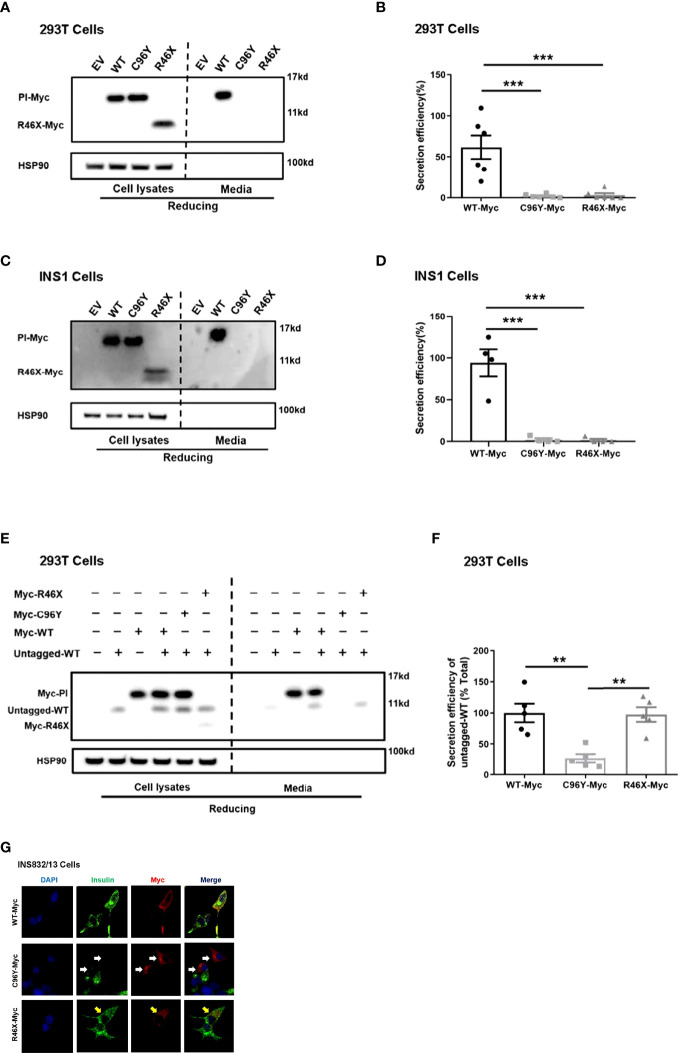
R46X impaired secretion efficiency of the mutant but did not affect secretion or production of bystander proinsulin-WT or production of endogenous insulin. **(A)** 293T cells were transiently transfected with empty vector (EV) or Myc-tagged plasmids encoding PPI-WT or mutants (C96Y-Myc, R46X-Myc). The culture media were changed at 24 h post-transfection. After an additional 24 h of incubation, both cell lysates (left panel) and media (right panel) were subjected to Western blotting under reducing conditions with anti-Myc antibody. **(B)** The secretion efficiency of proinsulin-WT and mutants (media/cells) in panel A was quantified and calculated. The results were shown as mean ± SEM from 6 independent experiments. *** indicates *p* < 0.001 compared to WT-Myc. **(C)** Similar experiments of panel A were performed in INS1 cells. **(D)** The secretion efficiency of PI-WT and mutants (media/cells) in panel C was quantified and calculated. The results were shown as mean ± SEM from 4 independent experiments. *** indicates *p* < 0.001 comparing to WT-Myc. **(E)** 293T cells were transiently co-transfected with untagged PPI-WT (WT-untagged) and Myc tagged PPI-WT or mutants (WT-Myc, C96Y-Myc and R46X-Myc). The culture media were changed at 24 h post-transfection. After an additional 24 h of incubation, both cell lysates (left panel) and media (right panel) were subjected to Western blotting under reducing conditions. **(F)** The co-expressed WT-untagged in cell lysates and media were quantified using ImageJ. The secretion efficiency of WT-untagged in panel E (media/cells) was calculated. The results were shown as mean ± SEM from 5 independent experiments. ** indicates *p* < 0.01. **(G)** INS832/13 cells were transfected with plasmids encoding Myc-tagged PPI-WT and mutants as indicated. At 48 h post-transfection, the cells were fixed and permeabilized. Confocal immunofluorescence microscopy was performed after double-stained with anti-Myc (red) and anti-insulin (green). White and yellow arrows indicated that β cells were successfully transfected to express C96Y and R46X, respectively.

At least 70 *INS* mutations in the coding region of the proinsulin domain have been reported to cause mutant *INS* gene induced diabetes of youth (MIDY). Most of them impair proinsulin folding and intracellular trafficking (2). We therefore firstly examined the secretion of R46X in transfected 293T cells and INS1 cells. An established proinsulin mutant PPI-C96Y (also called *Akita* proinsulin), which causes proinsulin misfolding and neonatal diabetes (19, 23), was used as a positive control. Although the total amount of proinsulin was comparable (**Figure 2A**, left panel), both R46X and C96Y failed to be secreted from the cells to the media in transfected cells (**Figures 2A, B**). This secretion defect was further confirmed in INS1 cells transfected with PPI-WT and mutants (**Figures 2C, D**). Theoretically, the amount of insulin expressed by a single allele is enough for control blood glucose *in vivo* (2). However, most diabetes causing *INS* mutants are heterozygous and present as autosomal dominant fashion, indicating trans-dominant negative effect of proinsulin mutants on bystander proinsulin-WT. We have previously reported that C96Y could interact with co-expressed WT proinsulin and prevent its ER export (26). We therefore asked whether this mechanism also applies for R46X mutant. We co-expressed WT-untagged with WT-Myc or mutants (C96Y-Myc and R46X-Myc) in 293T cells. Consistent with our previous observation, C96Y blocked secretion of co-expressed proinsulin-WT. In contrast, although R46X could not be secreted from the cells, it did not impair the secretion of bystander proinsulin-WT (**Figures 2E, F**). Next, we asked whether R46X could generate a dominant negative effect on insulin production from endogenous proinsulin in β cells. We transfected WT-Myc or mutants (C96Y-Myc and R46X-Myc) into INS832/13 cells. In the cells expressing C96Y-Myc (red), endogenous insulin (green) was significantly decreased compared with the neighboring control cells. However, although R46X itself displayed a diffused pattern within the cells, it did not appear to affect the insulin content of β cells (**Figure 2G**). Together, these results indicated that although the secretion efficiency of R46X was reduced, R46X did not affect secretion or production of bystander proinsulin-WT, nor did it impair insulin production from endogenous proinsulin.

### R46X Did Not Form Disulfide-Linked Proinsulin Complexes, nor Did It Interact With Proinsulin-WT

**Figure 3:**
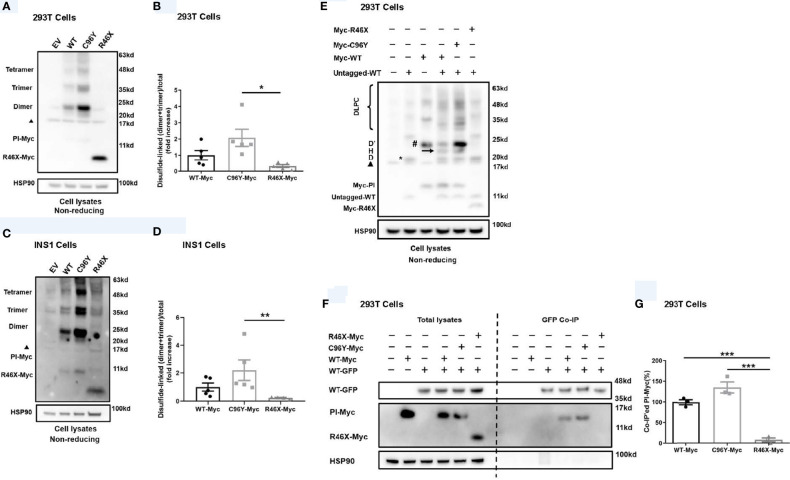
R46X did not form disulfide-linked proinsulin complexes itself, nor did it interact with co-expressed proinsulin-WT. **(A)** 293T cells were transiently transfected with empty vector (EV) or Myc-tagged plasmids encoding PPI-WT (WT-Myc) or mutants (C96Y-Myc, R46X-Myc). Cell lysates were subjected to Western blotting with anti-Myc antibody under non-reducing conditions. The non-specific bands at ~17 Kd were marked with a black triangle. **(B)** The disulfide-linked dimer and trimer under non-reducing conditions in panel A and total proinsulin under reducing condition in were quantified. The ratios (dimer + trimer/total proinsulin) were calculated and that of PI-WT was set to “1”. The results were shown as mean ± SEM from 4 independent experiments. * indicates *p* < 0.05. **(C)** Similar experiments to panel A were performed in INS1 cells. **(D)** The disulfide-linked dimer and trimer under non-reducing conditions in panel C and total proinsulin under reducing condition in were quantified. The ratios (dimer + trimer/total proinsulin) were calculated and that of proinsulin-WT was set to “1”. The results were shown as mean ± SEM from 4 independent experiments. ** indicates *p* < 0.01. **(E)** 293T cells were co-transfected with plasmids encoding untagged PPI-WT (WT-untagged) and Myc-tagged PPI-WT or mutants (WT-Myc, C96Y-Myc, and R46X-Myc). The monomer, disulfide-linked proinsulin dimers (D refers to homodimers formed by WT-Untagged, D’ refers to homodimers formed by Myc-tagged proinsulin, and H refers to heterodimers formed by Myc-tagged and untagged PI), and high-molecular-weight disulfide-linked proinsulin complexes (DLPC) were analyzed under non-reducing conditions. The non-specific bands at ~17 Kd were marked with a black triangle. **(F)** 293T cells were co-transfected with plasmids encoding GFP-tagged PPI-WT (WT-GFP) and Myc-tagged PPI-WT or mutants (WT-Myc, C96Y-Myc, and R46X-Myc) as indicated. At 48 h post-transfection, the cells were lysed and immunoprecipitated (IP) with anti-GFP antibody in co-IP buffer. The immunoprecipitants were resolved in 4%–12% NuPage gel and immuno-blotted with anti-proinsulin or anti-Myc antibodies under reducing conditions. **(G)** Myc-tagged PI in the total lysates and co-precipitated Myc-tagged PI (PI-Myc) were quantified using ImageJ. The percentages of co-precipitated Myc-tagged PI were calculated. The results were shown as mean ± SEM from 3 independent experiments. *** indicates *p* < 0.001.

In order to examine the effect of R46X on the folding of the mutant, we expressed Myc-tagged PPI-WT mutants in 293T cells and INS1 cells, and analyzed their oxidative folding under non-reducing conditions. Consistent with our previous reports (21, 27), we found that C96Y formed more misfolded disulfide-linked proinsulin complexes (DLPC, including dimer, trimer, tetramer, and high-molecular-weight proinsulin complexes) than that of WT. Surprisingly, however, unlike C96Y, R46X did not form DLPC itself in 293T cells (**Figures 3A, B**). Similar results were confirmed in INS1 cells transfected with Myc-tagged PPI-WT, C96Y, or R46X (**Figures 3C, D**). To examine whether R46X interacts with co-expressed PPI-WT, we co-expressed untagged PPI-WT with empty vector or Myc-tagged PPI-WT, or mutants (C96Y-MYC and R46X-Myc) in 293T cells. We found that, under non-reducing conditions, untagged PPI-WT and Myc-tagged PPI-WT could form disulfide-linked homodimers (marked as D and D’, respectively) as well as heterodimers (arrow, and also marked as “H”). Importantly, C96Y-Myc not only formed more disulfide-linked homodimers, but also formed heterodimers with co-expressed untagged PPI-WT (**Figure 3E**). On the contrary, R46X-Myc did not form homodimers by itself or form heterodimers with untagged PPI-WT (**Figure 3E**). To further confirm whether R46X interacted with proinsulin-WT in the ER, we performed co-immunoprecipitation experiments, in which GFP-tagged PPI-WT with Myc-tagged PPI-WT or mutants (C96Y-MYC and R46X-Myc) were expressed in 293T cells. We found that C96Y-Myc and WT-Myc could be co-immunoprecipitated by GFP-tagged PI-WT (WT-GFP). However, R46X could not be pulled down by PI-WT-GFP (**Figures 3F, G**), indicating that R46X did not interact with PI-WT in the ER. Furthermore, we explored the effect of R46X mutation on ER stress and β-cell survival. We used an established ER stress reporter (21, 25, 28, 29) Bip-promoter driven luciferase assay to evaluate ER stress response in both INS1 and Min6 cells expressing Myc-tagged PPI-WT or mutants. We found that unlike other *INS* mutations, R46X mutation did not induce significant increases in ER stress response (**Supplemental Figures 1A, B**). In order to examine whether R46X could affect β-cell survival, we have examined cleaved caspase 3 in Min6 cells expressing PPI-WT and mutants. Min6 cells treated with ER stress inducer thapsigargin was used as a positive control. We found that R46X did not increase cleaved caspase 3, a critical executioner of apoptosis, suggesting that it did not cause β-cell apoptosis (**Supplemental Figures 1C, D**). Together, those data indicate that R46X does not interact and affect co-expressed PI-WT and does not induce strong enough ER stress as other tested mutations do, which does not appear to lead to apoptosis at least based on the short-term *in vitro* assays in cell lines. The future *in vivo* experiments using transgenic/knock-in mice expressing R46X may bring more certainty regarding the effect of the mutant on β-cell stress responses and survival.

### R46X Causes an Inefficient Translocation Into the ER and Is Less Stable in the Cells

**Figure 4:**
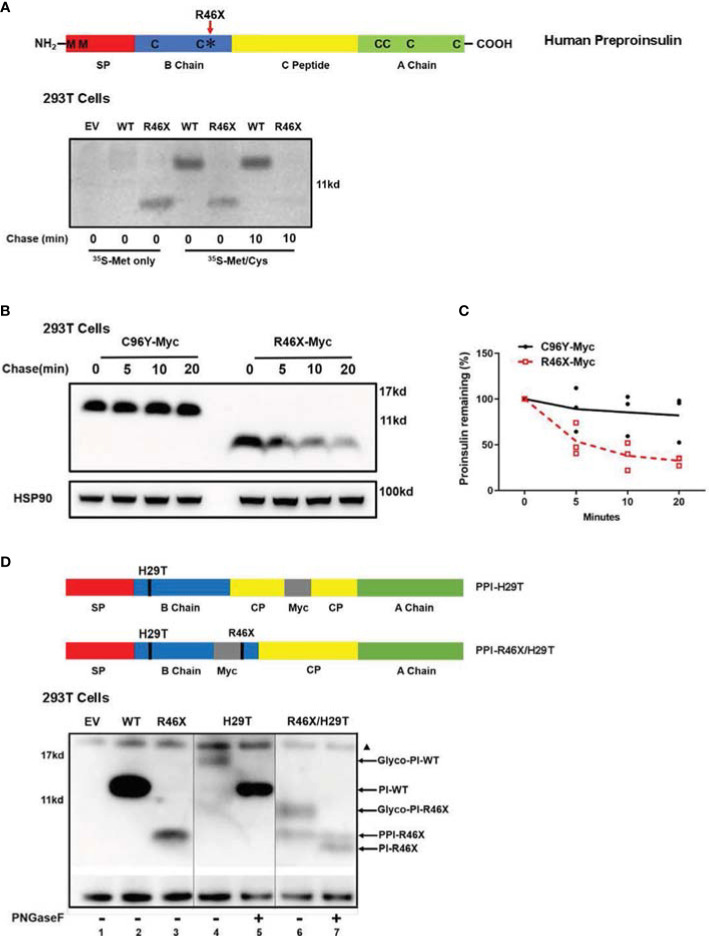
R46X causes an inefficient translocation into the ER and is less stable in the cells. **(A)** A sketch of PPI (upper panel): The signal peptide (SP, red), insulin B chain (blue), C peptide (yellow), and insulin A chain (green). Two methionines (M) locate only in the SP of human PPI. Six cysteines **(C)** present in both human PPI and PI. Bottom panel: 293T cells were transfected with plasmids encoding empty vector (EV), Myc-tagged PPI-WT, or R46X. At 48 h post-transfection, the cells were pulse-labeled with either pure ^35^S-Met or ^35^S-Cys/Met as indicated for 10 min followed by 0 or 10 min chase. The newly synthesized PPI and PI were immunoprecipitated with anti-Myc and analyzed by 4%–12% NuPage under reducing conditions. **(B)** 293T cells were transfected with plasmids encoding Myc-tagged PPI-C96Y or R46X. After 48 h transfection, cells were treated with 20 μg/ml cycloheximide (CHX) for the indicated times, and the cell lysates were collected and analyzed by Western blotting with anti-Myc. **(C)** The expression levels of C96Y and R46X in panel B were quantified with ImageJ. The results were presented as mean ± SEM from 4 independent experiments. **(D)** PPI mutants with N-linked glycosylation sites were shown in the upper panel. 293T cells were transfected with empty vector (lane 1) or plasmids encoding PPI-WT (lane 2), R46X (lane 3), H29T (lanes 4 and 5) or R46X/H29T (lanes 6 and 7). Forty-eight hours post-transfection, the cell lysates were treated with or without PNGaseF before analyzing using Western blotting under reducing conditions. The non-specific bands at ~17 Kd were marked with a black triangle.

To further investigate biological behavior of R46X, we performed metabolic labeling experiments. Since the two methionine residues present only in the signal peptide of PPI and six cysteine residues locate in the proinsulin domain (**Figure 4A**, upper panel), ^35^S-methionine (^35^S-Met only) can only label signal peptide attached PPI, but not proinsulin, whereas ^35^S-methionine/cysteine (^35^S-Met/Cys) mixture can label both PPI and proinsulin. As shown in **Figure 4A**, the newly synthesized PPI-WT and R46X were successfully detected in the cells labeled with ^35^S-Met/Cys. However, only R46X was detected in the cells labeled with ^35^S-Met only, suggesting that the signal peptide was still attached to the proinsulin domain of the R46X mutant (**Figure 4A**, the first five lanes of the bottom panel). Upon 10 min chase, a majority of the newly synthesized R46X was degraded (**Figure 4A**, the last two lanes of the bottom panel), suggesting that R46X was not stable in transfected 293T cells. Indeed, when we compared the stability of C96Y and R46X by Western blotting, we found that up to 60% of R46X was degraded in 20 min (**Figures 4B, C**).

At least two possible reasons can cause failure of signal peptide removal of PPI-R46X (**Figure 4A**). First, R46X caused inefficient translocation; therefore, the signal peptide cleavage site was not exposed to signal peptidase at the luminal side of the ER membrane. Second, R46X is successfully translocated into the ER; however, it cannot be efficiently cleaved by signal peptidase. To distinguish these possible causes, we mutated histidine to threonine at the 5th residue of proinsulin domain, generating an “N-X-T” *N-linked* glycosylation site (named as H29T). Consistent with our previous report (28), proinsulin WT was glycosylated as demonstrated by glycosylated proinsulin-WT that was deglycosylated upon the digestion with PNGase F (**Figure 4D**, lanes 4 vs. 5). However, R46X/H29T generated two bands (**Figure 4D**, lane 6). The upper band shifted down upon digestion of PNGase F, establishing that it entered the ER and acquired an *N-linked* glycan (**Figure 4D**, lane 7), whereas the lower band in the lane 6 did not shift upon treatment of PNGaseF, suggesting that it did not enter the ER lumen and was not glycosylated (**Figure 4D**. lanes 6 vs. 7). It is worth noting that, compared with R46X, more than 50% of the double mutant R46X/H29T did translocate into the ER and acquire an N-linked glycan (**Figure 4D**, lanes 3 vs. 6), suggesting that mutating histidine to threonine appeared to improve translocation efficiency of R46X. We have previously shown that increased PPI N-terminal charge gradient was favorable for translocation of PPI (6, 11). Therefore, the improvement of translocation efficiency of R46X/H29T was likely caused by mutation of histidine that increased charge gradient of PPI N-terminus. Altogether, these results established that R46X caused inefficient translocation and revealed an important role of proinsulin domain in determining translocation efficiency of PPI.

## Discussion

To date, at least 70 *INS* mutations have been reported to cause autosomal dominant diabetes in humans (2, 20, 30). Some additional mutations that disrupt transcription and translation of insulin gene due to *INS* deletion, promoter inactivation, and loss of translation initiation cause neonatal diabetes only when both *INS* alleles were affected (31), suggesting that one insulin gene allele may be sufficient to maintain normoglycemia under normal physiological conditions, although these recessive *INS* mutations are linked to an increased risk of diabetes (32, 33). Most dominant *INS* mutations are located in the proinsulin domain, impairing proinsulin oxidative folding and causing proinsulin misfolding in the ER (2, 16, 24, 34). Two main mechanisms underlie β-cell failure and diabetes caused by misfolded proinsulin mutants. First, misfolded proinsulin may accumulate in the ER, disturbing ER protein homeostasis, inducing chronic ER stress that may activate apoptotic pathway, leading to a decrease of β-cell mass (24, 35–37). Second, misfolded proinsulin can abnormally interact with co-expressed proinsulin-WT *via* proinsulin dimerization interface, impairing folding and the ER export of proinsulin-WT, decreasing insulin production, and leading to insulin-deficient diabetes (16, 21, 23, 28). More recently, a study shows that misfolded proinsulin may impair intracellular trafficking and processing of the precursor of insulin receptor, causing an impairment of insulin signaling in β cells, which may also contribute to β-cell failure caused by *INS* mutations (38). Interestingly, the clinical phenotypes associated with different *INS* mutations or even with same mutations range from severe neonatal diabetes to mild adult-onset diabetes, or some even without overt diabetes as the time of studies (2, 13, 16, 22, 39–42). In this report, we identified a novel nonsense *INS* mutation PPI-R46X associated with early-onset diabetes in two unrelated patients. Pedigree analysis showed a broad spectrum of clinical presentations among the members carrying R46X in the two families (**Figure 1** and **Tables 1**, **3**), suggesting that other factors that need to be further determined may contribute to actual clinical presentations associated with R46X. Functional studies showed that, although R46X failed to be secreted from the cells, unlike the mutations causing neonatal diabetes, R46X did not form DLPC, did not affect PPI-WT secretion and insulin production, and did not appear to induce strong ER stress or cause β-cell apoptosis (**Figures 2**, **3** and **Supplemental Figure 1**). In addition, R46X is less stable in the cells compared with C96Y (**Figures 4B, C**). All these data suggest that R46X may not be exposed to the oxidative ER environment. Indeed, two independent approaches demonstrated that, despite with an intact SP, R46X failed to be efficiently translocated into the ER (**Figures 4A, D**), highlighting a critically unrecognized role of proinsulin domain in efficient PPI translocation. Together, pedigree and functional analyses suggest that additional contributors may be required for developing diabetes in the patients carrying R46X. Further whole exome sequencing with linkage analysis among family members with or without R46X mutation, combined with functional studies, may shed light on better understanding what and how multiple genetic and/or environmental risk factors in concert with R46X lead to the development and progression of diabetes.

Preproinsulin is a small secretory protein with a suboptimal signal sequence that may not be efficiently recognized by the SRP and undergo SRP-dependent co-translational translocation across the ER membrane (7, 11). SRP-independent post-translational translocation is an important backup for efficient PPI translocation. However, the factors that determine the route and efficiency of PPI translocation remain to be further elucidated. PPI signal peptide mutations provide excellent molecular models and great opportunities to address these fundamental biological questions. To date, PPI signal peptide mutations that have been experimentally established to affect PPI translocation are PPI-R6C/H. These two mutations locate in the n-region of the PPI signal peptide, resulting in a decrease of charge gradient from PPI N-terminus to proinsulin domain. This charge gradient has been shown to play an important role in orientating PPI signal peptide in the Sec61 translocon during translocation (11), and it is more important for small secretory proteins including PPI for the post-translational translocation, because of the large secretory proteins that do not depend on the N-terminal positive charge for their translocation (6, 43), suggesting that the length of secretory proteins may also play a role in selecting the translocation pathway and efficiency. Indeed, R46X results in translating a very small truncated PPI, causing inefficient translocation. However, increasing a charge gradient in the N-terminus of R46X by mutating histidine to threonine at the 5th residue of proinsulin domain improves efficiency of R46X translocation by more than 50%, supporting the notion that both N-terminal positive charge and the length of PPI are important for efficient PPI translocation.

In summary, this study reveals that PPI translocation efficiency is affected not only by the natural property of its signal sequence, but also by its downstream of proinsulin domain. To the best of our knowledge, this is the first report to experimentally demonstrate that a mutation in the proinsulin domain causes PPI translocation inefficiency, which may underlie increased risk of diabetes-associated R46X. This study not only expends the list of *INS* mutations associated with diabetes, but also provides genetic and biological evidence underlying the regulation mechanism of PPI translocation.

## Data Availability Statement

The data sets generated during and analyzed during the current study are available from the corresponding author upon reasonable request. The data presented in this study are deposited in the GenBank repository, accession number PRJNA787369. The information will be accessible with the following link: https://www.ncbi.nlm.nih.gov/sra/PRJNA787369.

## Ethics Statement

The studies involving human participants were reviewed and approved by Tianjin Medical University General Hospital Ethics Committee (IRB2017-047-01 of 4 April 2017). Written informed consent to participate in this study was provided by the participants’ legal guardian/next of kin.

## Author Contributions

YY, HS, JH, LL, JW, TC, JZ, ZF, HG, KZ, and YX generated research data. JS, WF, YH, and XL contributed to discussion and reviewed/edited the manuscript. ML initiated and designed the research project, reviewed the data, and wrote the manuscript. ML is the guarantor of this work and, as such, had full access to all the data in the study and take responsibility for the integrity of the data and the accuracy of the data analysis. All authors contributed to the article and approved the submitted version.

## Funding

This work was supported by the National Natural Science Foundation of China (81620108004, 81830025, 81700699, 81870533, and 81900720), the Ministry of Science and Technology of China (2019YFA0802502), the Tianjin Municipal Science and Technology Bureau (17ZXMFSY00150 and 18JCYBJC93900), the Second Hospital of Tianjin Medical University Youth Program (2017YDEY19), the Scientific Research Project of Tianjin Educational Committee (2020KJ47), and the Fundamental Research Funds for the Central Universities of Peking Union Medical College (3332021083). The funders were not involved in the study design, collection, analysis, interpretation of data, the writing of this article or the decision to submit it for publication.

## Conflict of Interest

Author KZ is employed by RSR Tianjin Biotech Co., Ltd.

The remaining authors declare that the research was conducted in the absence of any commercial or financial relationships that could be construed as a potential conflict of interest.

## Publisher’s Note

All claims expressed in this article are solely those of the authors and do not necessarily represent those of their affiliated organizations, or those of the publisher, the editors and the reviewers. Any product that may be evaluated in this article, or claim that may be made by its manufacturer, is not guaranteed or endorsed by the publisher.
