# Supplementary material for: A Novel Nonsense INS Mutation Causes Inefficient Preproinsulin Translocation Into the Endoplasmic Reticulum
Source: Front Endocrinol (Lausanne). 2022 Jan 5;12:774634. doi: 10.3389/fendo.2021.774634 (PMC8769375; doi:10.3389/fendo.2021.774634)
Supplement: Supplementary file 1 [file DataSheet_1.docx]

Supplementary Material

**
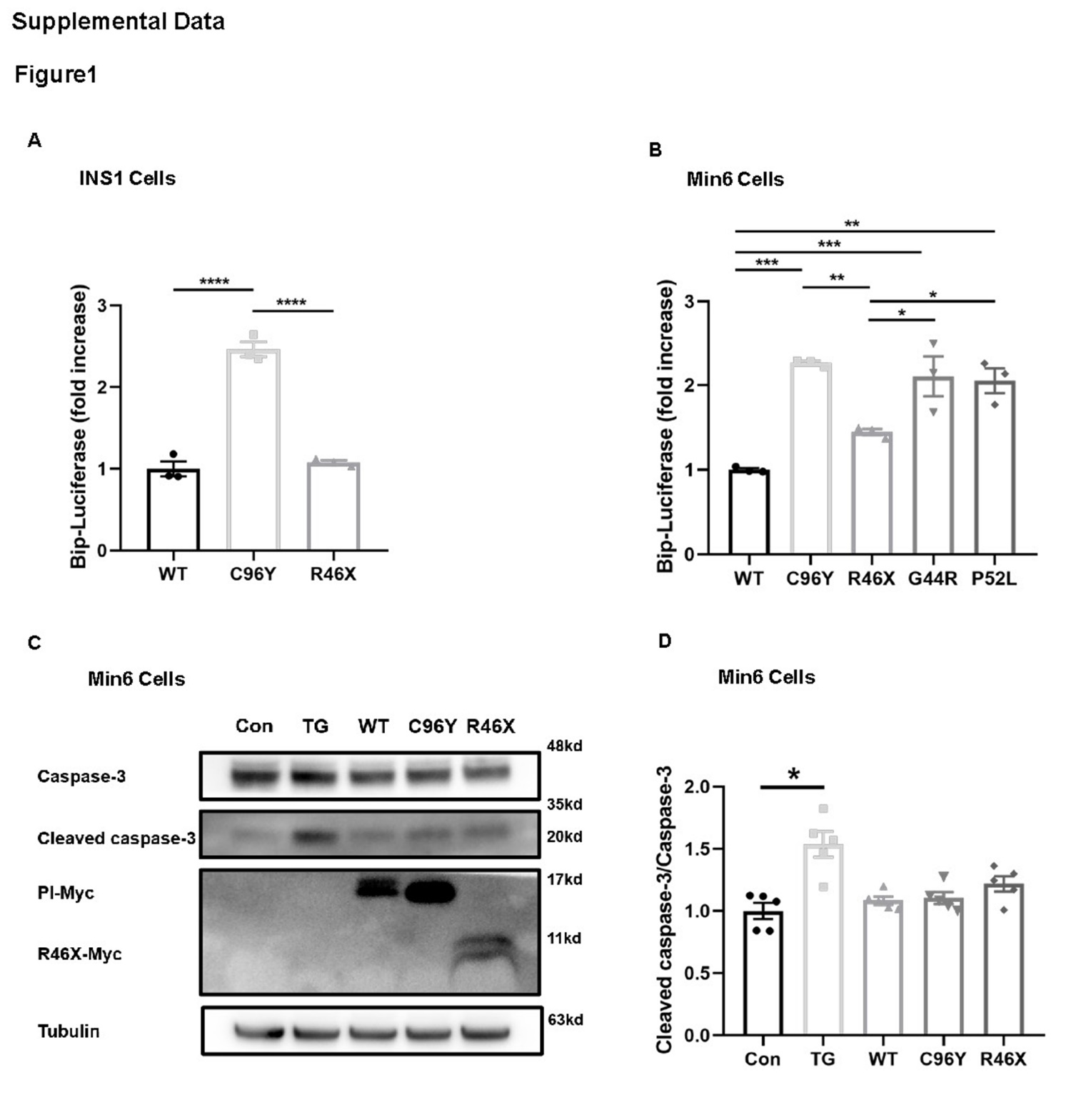
**

**Supplementary Figure 1.** **R46X did not appear to induce strong ER stress and did not increase cleaved caspase 3. A.** INS1 cells were plated into 24-well plates one day before the transfection and were triple-transfected with plasmids encoding PPI-WT or mutants (C96Y, R46X), pGL3-Bip (Bip promoter-firefly luciferase), or pRL-CMV (CMV driven renilla luciferase). A total of 1 μg plasmid DNA was transfected using lipofectamine 3000 for each well. The ratio of plasmids was 450:50:1. At 48h post-transfection, the cells were lysed and the luciferase activity was measured using the dual-luciferase reporter assay (Promega). The ratios of firefly/renilla luciferase were calculated. The relative activities of the Bip promoter in the cells transfected PPI-WT was set as “1”. Results were from three independent experiments. **** indicates p < 0.0001. **B.** Min6 cells were plated into 24-well plates one day before the transfection. The cells were triple-transfected with plasmids encoding PPI-WT or mutants (C96Y, R46X, G44R, P52L), pGL3-Bip, or pRL-CMV. A total of 1 μg plasmid DNA was transfected using lipofectamine 3000 for each well. The ratio of plasmids was 450:50:1. At 48h post-transfection, the cells were collected and the luciferase activity was measured as in supplemental Fig. 1A. The relative activities of the Bip promoter in the cells transfected PPI-WT was set as “1”. Results are from three independent experiments. * indicates p < 0.05, ** indicates p < 0.01, *** indicates p < 0.001. **C.** Min6 cells were plated into 24-well plates one day before the transfection. The cells were transfected with plasmids encoding PPI-WT and mutants. The cells transfected with empty vector was used as a negative control (Con). At 48 h post-transfection, the transfected cells were lysed and uncleaved and cleaved caspase 3 were examined by western blot using anti-caspase-3 and anti-cleaved caspase 3 antibodies (from Cell Signaling Technology, Danvers, MA, USA), respectively. The cells treated with ER stress inducer 1μM thapsigargin (TG) for 4 hours was used as a positive control. **D.** Uncleaved caspase-3 and cleaved caspase-3 in supplemental Fig. 1C were quantified using Image J. The ratios of cleaved caspase-3/caspase-3 were calculated. The results were shown as mean ± SEM from 5 independent experiments. * indicates p < 0.05.
